# Neural Basis and Motor Imagery Intervention Methodology Based on Neuroimaging Studies in Children With Developmental Coordination Disorders: A Review

**DOI:** 10.3389/fnhum.2021.620599

**Published:** 2021-01-22

**Authors:** Keisuke Irie, Amiri Matsumoto, Shuo Zhao, Toshihiro Kato, Nan Liang

**Affiliations:** ^1^Cognitive Motor Neuroscience, Department of Human Health Sciences, Graduate School of Medicine, Kyoto University, Kyoto, Japan; ^2^School of Psychology, Shenzhen Key Laboratory of Affective and Social Neuroscience, Shenzhen University, Shenzhen, China; ^3^Rehabilitation of Developmental Disorders, Department of Human Health Sciences, Graduate School of Medicine, Kyoto University, Kyoto, Japan

**Keywords:** developmental coordination disorder, neuroimaging, brain, motor imagery, methods

## Abstract

Although the neural bases of the brain associated with movement disorders in children with developmental coordination disorder (DCD) are becoming clearer, the information is not sufficient because of the lack of extensive brain function research. Therefore, it is controversial about effective intervention methods focusing on brain function. One of the rehabilitation techniques for movement disorders involves intervention using motor imagery (MI). MI is often used for movement disorders, but most studies involve adults and healthy children, and the MI method for children with DCD has not been studied in detail. Therefore, a review was conducted to clarify the neuroscientific basis of the methodology of intervention using MI for children with DCD. The neuroimaging review included 20 magnetic resonance imaging studies, and the neurorehabilitation review included four MI intervention studies. In addition to previously reported neural bases, our results indicate decreased activity of the bilateral thalamus, decreased connectivity of the sensory-motor cortex and the left posterior middle temporal gyrus, bilateral posterior cingulate cortex, precuneus, cerebellum, and basal ganglia, loss of connectivity superiority in the abovementioned areas. Furthermore, reduction of gray matter volume in the right superior frontal gyrus and middle frontal gyrus, lower fractional anisotropy, and axial diffusivity in regions of white matter pathways were found in DCD. As a result of the review, children with DCD had less activation of the left brain, especially those with mirror neurons system (MNS) and sensory integration functions. On the contrary, the area important for the visual space processing of the right brain was activated. Regarding of characteristic of the MI methods was that children observed a video related to motor skills before the intervention. Also, they performed visual-motor tasks before MI training sessions. Adding action observation during MI activates the MNS, and performing visual-motor tasks activates the basal ganglia. These methods may improve the deactivated brain regions of children with DCD and may be useful as conditioning before starting training. Furthermore, we propose a process for sharing the contents of MI with the therapist in language and determining exercise strategies.

## Introduction

Developmental coordination disorder (DCD) manifests as “clumsiness and slowness or inaccuracy of motor skills and defective acquisition and performance of coordination skills, which interfere with activities of daily living.” The prevalence is 5–6% in children aged 5–11 years, and the sex ratio ranges from 2:1 to 7:1 (male:female; American Psychiatric Association, 2013). Various subtypes of motor problems have been reported and commonly include issues related to motor skills such as balance, coordination, and writing (Nakai et al., 2011; Vaivre-Douret et al., 2011). Underdeveloped motor skills make it difficult to perform the basic movements required for daily activities (Wilson et al., 2013; Adams et al., 2016a). Furthermore, problems associated with DCD extend to exercise-related activities as well as other aspects. For example, reduced participation in play and group sports causes physical problems such as weakness and obesity (Watkinson et al., 2001; Mandich et al., 2003; Cairney et al., 2005). Self-esteem and self-affirmation may be impaired, and secondary disorders such as depression and anxiety-related mental disorders have also been recognized (Poulsen et al., 2008; Lingam et al., 2012; Missiuna et al., 2014; Caçola, 2016; Cairney et al., 2016).

DCD can occur alone or with other diseases and disorders. In particular, its coexistence with attention-deficit/hyperactivity disorder (ADHD), termed “deficit of attention, motor control, and perception syndrome”, is high (Fliers et al., 2008; Díaz-Lucero et al., 2011); moreover, it is reported that more than 80% of individuals with coexisting autism spectrum disorder (ASD) experience significant problems in daily life situations (Green et al., 2009; Van Waelvelde et al., 2010). Impaired spatial grasping ability and visual and motor perception may underlie these comorbidities, but the common neurological basis has not been clarified.

The exercise-related problems of children with DCD rarely resolve spontaneously with age. They often persist in adolescence and adulthood (Zwicker et al., 2012b; Bo and Lee, 2013), and may further promote secondary disabilities given the lack of proper intervention (Cantell et al., 1994). Therefore, some form of support becomes necessary. Various programs have been implemented for exercise support to children with DCD, and some short-term results have been reported (Yu et al., 2018). It is known that training that simply involving correcting inaccurate coordination is not always effective, and nowadays the usefulness of a task-oriented approach, in which the child finds multiple solutions and selects the most desirable one, has been suggested (Smits-Engelsman et al., 2018). However, a systematic review and meta-analyses published between 1996 and 2012, judged to be of low quality in a report, questioned the quality of the evidence in this regard and the effectiveness of such interventions (Miyahara et al., 2017, 2020). In other words, there are very few rigorously planned and verified studies and corresponding reviews regarding interventions for children with DCD, and there is currently no evidence to prove that these interventions improve outcomes. In recent years, attempts have been made to develop international guidelines for DCD (Blank et al., 2019), and interventions involving task-oriented approaches (Ward and Rodger, 2004), and neuromotor task training (Ferguson et al., 2013) are recommended, indicating that these are considered effective. Besides, from a novel perspective, interventions using motor imagery (MI) have also been reported. Since MI simulates in the brain without actually exercising, it is less likely to cause exercise errors and may be useful as a pre-training condition for children with DCD. A point to be noted while carrying out exercise image intervention is that the intervention method differs depending on factors such as the age and condition of the target individual and the type of exercise. To address this issue, Schuster et al. (2011) analyzed systematic MI training sessions (MITS) and reported the details of successful MI intervention techniques. However, most of the studies analyzed involved interventions for adults, and only two involved interventions for children up to 9 years of age. Furthermore, both studies involved interventions for healthy children. Therefore, it is necessary to investigate the methodology of MI intervention for children with DCD that is currently being conducted and integrate it with the results of brain imaging studies to derive effective intervention methods.

Various studies using brain functional imaging to study the pathophysiology of DCD have also been conducted. Based on functional MRI (fMRI) studies using hand movement tasks, compared to children with typical development (TD), children with DCD were found to have lower activation in the middle frontal gyrus (MFG), superior frontal gyrus (SFG), cerebellum, supramarginal gyrus (SMG), and inferior parietal lobules (IPLs; Fuelscher et al., 2018). A study focusing on the mirror neuron system (MNS), including the inferior frontal gyrus (IFG), premotor cortex (PMC), IPL, and superior temporal sulcus, has also been reported (Reynolds et al., 2015b; Lust et al., 2019). In 2016, a critical review of previous MRI studies was published and concluded that the neural bases in children with DCD included the frontal lobe, parietal lobe, basal ganglia, and cerebellum (Biotteau et al., 2016). As mentioned above, knowledge of DCD’s neural basis and network abnormalities has been accumulated, but few studies have mentioned intervention methods based on these studies. Therefore, we focused on MI, which is one of the neurological rehabilitation, and aimed to derive an effective intervention method for children with DCD based on the results of neuroimaging studies.

The purpose of this study was to examine MI interventions for children with DCD based on neuroimaging studies and to propose new methods. Therefore, we planned to carry out two reviews. One reviewed MRI articles up to 2020 and summarized the latest information on how the neural bases and networks of children with DCD differ from those of children with TD. The other was to summarize the MI intervention methods used for children with DCD.

## Materials and Methods

A comprehensive search was completed in the databases Medline, CINAHAL, AMED, and The Cochrane Library.

### Neuroimaging Studies

The search strategy used MeSH terms and text words for (“child” or “child, preschool,” or “pediatric”) and (“motor skills disorders” or “developmental coordination disorder” or ‘DCD’) and (“Magnetic Resonance Imaging” or “functional connectivity” or “neural pathways”) in August 2020. Brain function analyses using MRI for children with DCD involve the following: (1) fMRI; (2) diffusion tensor imaging (DTI); or (3) voxel-based morphometry (VBM). Exclusion criteria were: (1) adult studies or preterm children; (2) review and meta-analysis literature; (3) cerebral palsy; and (4) dysgraphia.

### fMRI

Based on the blood-oxygenation-level-dependent effect (Kim and Ogawa, 2012), somatosensory sensations, such as visual, auditory, tactile, taste, and olfactory sensations, can be identified using an MR device by analyzing the increase in blood flow associated with brain activity and identifying the activation site. Similarly, it is possible to understand which part of the brain is active when exercise or cognitive stimulation is applied. It is also possible to investigate neural networks, language, memory, emotion, attention, and brain plasticity. Studies have also focused on resting-state MRI (Buzsákim and Draguhn, 2004) because neural activity in the brain fluctuates with a certain frequency band even in the resting state (Raichle, 2011). With this method, the subject is taught to be at rest by keeping their eyes closed or gazing at a fixed point. In many cases, the measured spontaneous volatility of the blood-oxygenation-level-dependent signal is used to assess the degree of correlation between the neural activities of distant regions. This synchronization between brain regions is called functional connectivity, and a functional index or a network index (the default mode network) based on it has been proposed as a new biological index (Mohan et al., 2016).

### DTI

Diffusion-weighted images are used as the basis for calculations in DTI. This method has been used to evaluate the diffusivity of water molecules in the brain, where the direction of diffusion of water molecules is determined by the direction of nerve fiber conduction (Basser et al., 1994). Two types of indices are obtained from DTI: fractional anisotropy (FA), which represents the degree of diffusion anisotropy, and apparent diffusion coefficient, which represents the apparent magnitude of diffusion. It is also possible to observe the positional relationship between the nerve fibers in the body tract, sensory tract, visual axis, and lesion site.

### VBM

After anatomical standardization/tissue fractionation (for demarcation into gray matter, white matter, and cerebrospinal fluid space), image analysis of brain morphology is performed pixel-by-pixel based on the image database of the normal brain and specific factors (sex, age, lifestyle habits, neuropsychiatric disorders; Ashburner and Friston, 2000).

### Neurorehabilitation Studies (MI)

The search strategy used MeSH terms and text words for (“child” or “child, preschool,” or “pediatric”) and (“developmental disabilities” or “motor skills disorders” or “developmental coordination disorder” or ‘DCD’) and (“mental imagery” or “mental practice” or “mental training” or “mental rehearsal” or “mental movements” or “eidetic imagery” or “visual imagery” or “guided imagery” or “motor imagery”). The inclusion criteria were: (1) any design of quantitative intervention studies with a focus on imaging movements; (2) studies that included children with DCD; and (3) study intervention that focused on motor skill, performance, or strength improvement. Exclusion criteria were: (1) mental practice not related movements; and (2) mental practice without physical exercise. MITS was classified based on the 17 elements of the PETTLEP (physical, environment, timing, task, learning, emotion, and perspective; Holmes and Collins, 2001) approach-based MITS reported by Schuster et al. (2011; Supplementary Table 2). The Physiotherapy Evidence Database list was used to evaluate RCTs and assign a maximum score of 10 points; (Maher et al., 2003). An RCT is a study in which people are allocated at random (by chance alone) to receive one of several clinical interventions. One of these interventions is the standard of comparison or control. The control may be a standard practice, a placebo, or no intervention at all. For the case series experimental design, the 11-point Single Case Experimental Design scale was used (Tate et al., 2008). All studies were rated by the first author based on detailed rating guidelines. Studies received one point for each fulfilled methodological criterion on the respective rating list. The higher the achieved score, the better the study quality.

## Results of Neuroimaging Studies

**Figure 1:**
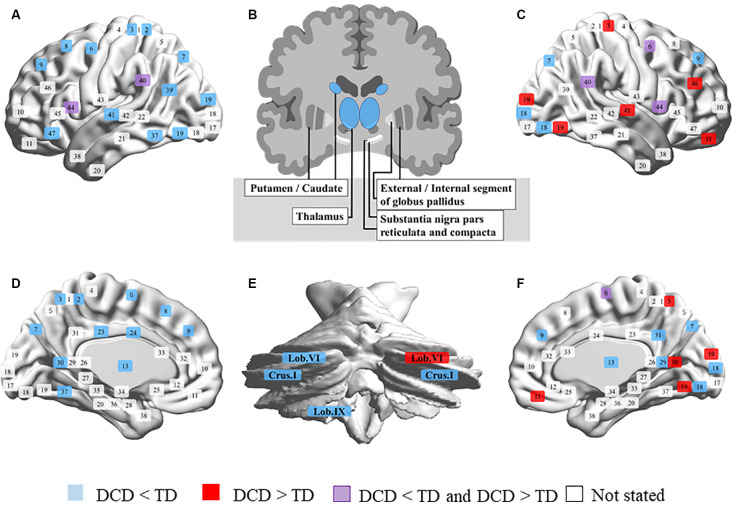
Comparison of brain activity in developmental coordination disorder (DCD) and typical development (TD). **(A)** Left outside of the sagittal plane. **(B)** Coronal plane of the basal ganglia. **(C)** Right outside of the sagittal plane. **(D)** Left inside of the sagittal plane. **(E)** Coronal plane of the cerebellum. **(F)** Right inside of the sagittal plane. Brodmann area (BA) 1, 2, 3, postcentral gyrus; 4, precentral gyrus; 5, superior parietal lobule; 6, premotor cortex and supplementary motor cortex; 7, superior parietal lobule; 8, frontal eye fields; 9, dorsolateral prefrontal cortex; 10, anterior prefrontal cortex; 11, 12, superior frontal gyrus; 13, insular cortex; 17, primary visual cortex; 18, secondary visual cortex; 19, associative visual cortex; 20, inferior temporal gyrus; 21, middle temporal gyrus; 22, superior temporal gyrus; 23, 24, 28–33, cingulate cortex; 25, subgenual area; 26, ectosplenial portion of the retrosplenial region of the cerebral cortex; 27, piriform cortex; 34, dorsal entorhinal cortex; 35, 36, perirhinal cortex and ectorhinal area; 37, fusiform gyrus; 38, temporal pole; 39, angular gyrus; 40, supramarginal gyrus; 41, 42, primary auditory cortex; 43, primary gustatory cortex; 44, pars opercularis, part of the inferior frontal gyrus; 45, pars triangularis, part of the inferior frontal gyrus; 46, dorsolateral prefrontal cortex; 47, pars orbitalis, part of the inferior frontal gyrus.

The neuroimaging review included 20 magnetic resonance imaging studies. In all studies, children with DCD experienced problems in their daily lives and were mostly assessed using the Movement Assessment Battery for Children (one study had unclear criteria and the other used the Bruininks-Oseretsky Test of Motor Proficiency). Most participants were 7–12 years old, but some studies included those up to 17 years of age. Participants performed various tasks during fMRI, such as go/no-go, tracking, fine-motor, trial-tracking, motor response, finger sequencing, and hand clenching tasks and observing, executing, and imitating a finger sequence, finger tapping sequence, and finger adduction/abduction. Others were conducted in the resting state, or the tasks were not listed. MRI was conducted at a magnetic flux density of 1.5 T in only one study and of 3.0 T in others. The details of the results are summarized in Supplementary Table 1. We have also depicted the results in Figure 1 to clearly show the differences in brain activation in DCD and TD. If brain activation were DCD > TD, the corresponding Brodmann area (BA) number is displayed in red. On the contrary, if brain activation were DCD < TD, it is displayed in blue. When reports were inconclusive (DCD > TD or DCD < TD), they are displayed in purple. Regions not mentioned are shown in white. BrainNet Viewer was used to creating the figure^1^ (Xia et al., 2013).

### fMRI Results

It was clear that the activation of the left brain was broadly reduced in children with DCD, while that in parts of the right brain was higher than in children with TD (Figure 1). The results are summarized below according to the sites and analysis types.

### Frontal Lobe

The left medial frontal gyrus (BA6), SFG (BA8), bilateral SFG (BA9), right dorsolateral prefrontal cortex (BA9), IFG (BA9), MFG (BA9), and left IFG (BA47) had lower activation in children with DCD than in children with TD. Conversely, the right lateral orbitofrontal cortex (BA11) and MFG (BA46) are more active in children with DCD (Caeyenberghs et al., 2016). The right precentral gyrus and medial frontal gyrus (BA6) showed a decline in activation in two studies (Reynolds et al., 2015a, 2017), but in the study by Zwicker et al. (2010) high activation was reported. In the pars opercularis of the IFG (BA44), children with DCD had lower activation during imitation and higher activation during observation than those in children with TD (Licari et al., 2015; Reynolds et al., 2015a).

### Parietal Lobe

In the left postcentral gyrus (BA2, 3), superior parietal lobe (BA7), bilateral precuneus (BA7), and left precuneus (BA39), children with DCD showed lower brain activation than children with TD. Conversely, the right postcentral gyrus (BA3) is activated to a greater extent in children with DCD. The bilateral IPL (BA40), SMG (BA40), and temporoparietal junction (BA40) are less activated those in children with DCD (Kashiwagi et al., 2009; Zwicker et al., 2011; Debrabant et al., 2013), while a study by Zwicker et al. (2010) reported high activation of the left IPL and right SMG.

### Temporal Lobe

In the left fusiform gyrus (BA37), superior temporal gyrus (BA41), and transverse temporal gyrus (BA41), children with DCD have lower brain activation than children with TD (Zwicker et al., 2011; Debrabant et al., 2013). Conversely, the right superior temporal gyrus (BA41) is more active than in children with DCD (Zwicker et al., 2010).

### Occipital Lobe

In the right lingual gyrus (BA18) and left middle temporal gyrus (MTG, BA19), children with DCD have lower brain activation than children with TD (Zwicker et al., 2011; Reynolds et al., 2015a). In the right lingual gyrus (BA19), activation was shown to be higher those in children with DCD (Zwicker et al., 2010).

### Limbic System and Islands

In the limbic system and islands, the bilateral insula (BA13), left cingulate gyrus (BA23, 24), right posterior cingulate (BA29), left parahippocampal gyrus (BA30), posterior cingulate (BA30), and right precuneus (BA31), children with DCD were shown to have lower brain activation than children with TD (Reynolds et al., 2015a, 2019). Conversely, the right parahippocampal gyrus (BA30) was shown to be more active in some children with DCD (Zwicker et al., 2010).

### Basal Ganglia and Cerebellum

In the basal ganglia, children with DCD have been shown to have lower brain activation in the bilateral thalamus and caudate than children with TD (Reynolds et al., 2019). In the cerebellum, bilateral cerebellar crus I, and left cerebellar lobules VI and IX, children with DCD have lower brain activation than children with TD (Zwicker et al., 2011; Debrabant et al., 2013). Conversely, the right cerebellar lobule VI has higher activation than in children with DCD (Zwicker et al., 2010).

### Connectivity

The results were summarized in Supplementary Table 1. Querne et al. found that, compared to children with TD, children with DCD had weaker connections between the right middle frontal cortex (BA46) and anterior cingulate cortex (BA32) and the middle frontal cortex (BA46) and inferior parietal cortex (BA40). On the other hand, the connection between the bilateral anterior cingulate cortex (BA32) and inferior parietal cortex (BA40) and the left middle frontal cortex (BA46) and inferior parietal cortex (BA40) is stronger in children with DCD (Querne et al., 2008). Mcleod et al. used resting-state fMRI analysis to show that the connection between the left M1 and bilateral IFG, insular cortex, superior temporal gyrus and caudate, right FOC, SMG, nucleus accumbens, pallidum, and putamen was weaker in children with DCD (McLeod et al., 2014). Also, they investigated the association of the sensorimotor cortex (SM1) with the basal ganglia and cerebellum, and in TD, the right thalamus and left cerebellar lobe V were found to be more strongly associated with the right SM1 than the left. However, in children with DCD, the left thalamus and right cerebellar lobe V were more strongly associated with the left SM1 than with the right. The right putamen was more strongly associated with the right SM1 than with the left in the TD group. However, in children with DCD, no strong intrahemispheric connections with the motor cortex were found in the right putamen, which was equally well-connected to the left and right SM1 (McLeod et al., 2016). Also, Rinat et al. (2020) showed that the connection between bilateral SM1 and posterior cingulate cortex (PCC; BA23, BA31) and precuneus (BA7, BA31), SM1 and left posterior middle temporal gyrus (pMTG) was weaker in children with DCD than that in children with TD.

### DTI Study Results

Zwicker et al. (2012a) showed that children with DCD had lower mean diffusivity in the corticospinal tract than children with TD. Furthermore, posterior thalamic radiation also decreased axial diffusivity. Langevin et al. (2014) reported that in the bilateral superior posterior parietal and left superior longitudinal fasciculus III, FA in children with DCD was lower than in those with TD. According to a report by Debrabant et al. (2016) the FA of the left retrolenticular limb of the internal capsule was lower in children with DCD compared to those with TD, while the radial diffusivity was increased; the same trend was observed on the right. They used a predictive statistical model to show that the cerebellum lobule VI and the right parietal superior gyrus are the most effective for distinguishing children with DCD from children with TD. Also, a study by Brown-Lum et al. (2020) which investigated the entire brain, showed a decrease in FA of the cerebral peduncle, superior cerebellar peduncle, external capsule, and splenium of the corpus callosum. In the corticospinal tract, cerebral peduncle, posterior thalamic radiation at the retrolenticular part of the internal capsule and external capsule, axial diffusivity was also reduced.

### VBM Study Results

Reynolds et al. (2017) reported that, compared to children with TD, children with DCD had significantly greater gray matter volume, which decreased to the right in the right SFG (BA6) and right MFG (BA6, 8).

## Discussion (Neuroimaging Studies)

Regarding the neural bases of DCD, a review of fMRI studies published in 2016 referred to the frontal lobe, parietal lobe, basal ganglia, and cerebellum (Biotteau et al., 2016). Our review indicates decreased activity in the bilateral thalamus decreased connectivity of the SM1 and left posterior middle temporal gyrus, bilateral posterior cingulate cortex, precuneus, cerebellum, and basal ganglia, loss of connectivity superiority in the above regions. Furthermore, reduction of gray matter volume in the right SFG and MFG and lower FA and axial diffusivity in regions of white matter pathways were found in DCD.

The thalamus plays an important role in relaying sensory information (visual, auditory, somatosensory, etc.) to the cerebral cortex. Somatosensory information is sent to the SM1 and the IPL *via* the thalamus, the efferent copies of exercise are integrated, and the exercise program is modified. Also, the thalamus is involved in selective visual spatial attention and relays attentional feedback to the visual cortex (Saalmann et al., 2012; Zhou et al., 2016). Hypothalamic inactivity leads to the inhibition of this sensory information, which may be related to the problems of motor planning and visuospatial cognition of children with DCD.

It has been suggested that the MTG is involved in the visual and auditory perception of tools and in tool movement in cooperation with the bilateral fusiform gyrus and the left parietal lobe (Assmus et al., 2007; Xu et al., 2016; Tomasello et al., 2017). Also, the MTG has been reported to play a role in the recognition of semantic actions, the expression of such actions, action monitoring during the performance, and comparison of sensory input and sensory prediction (Kalénine et al., 2010; Wallentin et al., 2011; Davey et al., 2015; Aue et al., 2019; van Kemenade et al., 2019); it is also thought to combine sensorimotor knowledge of meaningful behavior. When these observations are collectively interpreted, it is clear that MTG plays an important role in behavior-related knowledge and interpretation. The problem of gripping and using tools in children with DCD may be related to the inactivation of MTG. The PCC is involved in many cognitive functions, such as visual processing, motor performance (Field et al., 2015), visual space navigation (Bzdok et al., 2015), and decision-making (Heilbronner et al., 2011). The precuneus is involved in self-related processes such as retrieval of autobiographical and episodic memory, visual-spatial processing, and MI. Many studies have shown the involvement of the PCC and precuneus in various aspects of visual-spatial processing. Visuospatial abilities are associated with DCD, and decreased connectivity with the SM1 may be associated with diminished motor control that is dependent on visuospatial information (Tsai et al., 2009, 2012).

Strong functional connections in the thalamus on the ipsilateral side of the right brain and the cerebellum V on the contralateral side were observed in TD. On the other hand, children with DCD had strong functional connections to the thalamus in the left side of the brain and the contralateral cerebellum V. This observation was first reported by McLeod et al. (2016). One possibility is that children with TD have to mitigate the non-dominance of their left hand to perform tasks with both hands smoothly. Children with DCD have stronger functional connections to compensate for clumsiness due to sensory-motor disorder of the right hand. Besides, the children with TD and ADHD had a strong connection of the putamen on the ipsilateral side with the right SM1, whereas children with DCD strong connections on both the left and the right. In a previous study, children with DCD also showed a decrease in the diffusivity of the corticospinal tract (Zwicker et al., 2012a), suggesting that the unilateral significance of the dominant hand seen in TD is low.

Decreased gray matter volume in the right premotor and frontal lobes is associated with DCD-related dysfunctions, such as those related to working memory (Tsai et al., 2012), motor planning and performance, and attention (Tsai et al., 2009). The MTG is involved in motor control (Hanakawa et al., 2008) and contributes to decision-making and inhibitory control (Garavan et al., 1999; Talati and Hirsch, 2005); problems with these brain functions may be associated with motor control issues and behavioral consequences of poor accuracy or efficiency in children with DCD (Wilson et al., 2002; Adams et al., 2014; Reynolds et al., 2015b). Furthermore, the relationship between movement and brain function during motor control in children with DCD has been confirmed based on both cerebral blood flow in fMRI and event-related potential in electroencephalography (Zwicker et al., 2010, 2011; Pangelinan et al., 2013).

The corticospinal tract is an extensive network of projected white matter pathways that connect the primary motor cortex to the spinal cord *via* the corona radiata, internal capsule hind limbs, and cerebral peduncle. The posterior thalamic radiation at the retrolenticular part of the internal capsule and external capsule is another network of projected white matter tracts associated with sensory and motor processing. Previous studies have also shown that children with DCD have low FA in these areas (Zwicker et al., 2012a). Brown-Lum et al. (2020) found that children with DCD also had low FA in the cerebellum pathway by examining the entire brain. These pathways enter and exit the spinal cord, pons, and cerebral cortex, and cerebellum, helping to improve motor movements, learn new motor skills, and balance proprioceptive information into a posture (Keser et al., 2015). This finding complements the findings from functional MRI studies that showed inactivation of the cerebellar and mural regions in children with DCD compared to children with TD.

Focusing on the red and blue color in Figure 1, it is interesting that children with DCD have less extensive activation of the left brain than those in children with TD, and that activation in parts of the right brain (BA3, 11, 19, 30, 41, 46, cerebellar lobule VI) was enhanced. It has been pointed out that children with DCD often have problems with cross-modal information processing involving visual space recognition, kinesthetic perception, and matching of vision and proprioceptive sensation (Wilson and McKenzie, 1998; Schoemaker et al., 2001; Gomez and Sirigu, 2015). This activation may be a result of trying to compensate for the problem of sensory integration by visual space processing. It can also be interpreted that interhemispheric inhibition (IHI) occurs due to repeated high activity in the right hemisphere. However, since there are very few reports on activation at this time, careful discussion regarding this aspect is still needed. In rehabilitation interventions for children with DCD, it is necessary to aim at reconstructing brain function, and MI is one of the intervention methods. MI is defined as mentally evoking a certain motion and is a method used in multiple fields, such as sports, education, psychology, and rehabilitation (Caeyenberghs et al., 2009; Cumming and Ramsey, 2009). Especially in the area of rehabilitation, randomized controlled trials (RCTs) have shown their effectiveness for neurorehabilitation after stroke (Page et al., 2001; Liu et al., 2004). With the recent development of brain science methods, the neural basis of MI is becoming clear. Previous studies have repeatedly reported that brain activity similar to that at the time of motor execution occurs during MI (Zabicki et al., 2017). It has been reported that MI activates the bilateral PMC, supplementary motor area, dorsal and ventral PMCs, superior and inferior parietal lobules, basal ganglia (putamen), cerebellum (lobule VI), and left cingulate gyrus (Hardwick et al., 2018). Some of the brain areas that are activated by MI overlap with the neural base of DCD. Also, studies using transcranial magnetic stimulation have shown plastic changes due to mental practice and an increase in the excitability of the M1 during MI (Kasai et al., 1997; Stinear et al., 2006; Avanzino et al., 2015).

## Results of Neurorehabilitation Studies

In total, three studies by Australian and European groups published in Wilson et al. (2002, 2016), and Adams et al. (2017), and one protocol listed in Adams et al. (2016b) were included in the literature review. Concerning study design, two studies were RCTs, while one was a case series. Study quality was rated on a 10-point scale for RCTs and an 11-point scale for the case series. All interventions were for children with DCD between the ages of 7 and 12 years. The extracted information is summarized in detail in Table 1.

**Table 1:** Overview of extracted descriptive previous studies.

References	Intervention duration (days)	Study design	Groups	Patients	Participants	Gender	Age	Training task	Measurement	Results	Quality rating
Wilson et al. (2002)	5	RCT	3	54	DCD	NSt	7–12	Catching a tennis ball, throwing a tennis ball, striking a softball, jumping to a target using a two-leg take-off, balancing a ball on a bat while walking, placing objects using a formboard.	MABC	↑	3/10
Wilson et al. (2016)	5	RCT	3	36	DCD	NSt	7–12	Catching a tennis ball, throwing a tennis ball, striking a softball, jumping to a target using a two-leg take-off, balancing a ball on a bat while walking, placing objects using a formboard.	MABC	↑	3/10
Adams et al. (2017)	9	CCS	2	8	DCD	Both	7–12	Running and playing tag, throwing and catching a ball, hopping and playing hopscotch, jumping (almost others rope skipping), bicycling, playing baseball, playing tennis, writing, eating with cutlery.	MABC-2	↑	5/11

*NSt, not stated; M-ABC, movement assessment battery for children; M-ABC-2, movement assessment battery for children–second edition; ↑, positive change*.

The MITS factors for all MI interventions are summarized in Table 2. MI was performed in individual sessions and added or embedded before, between, or after physical practice (PP). MI sessions were supervised by a research assistant or therapist. The position of the participants during MI was task-specific. Participants received acoustic and visual MI instructions, which were mainly standardized and pre-recorded. The perspective used during MI practice was chosen from both internal and external viewpoints. The MI used kinesthetic as well as visual modes, and MI interventions were mainly investigated with respect to motor-focused tasks. All interventions involved watching a video of the movement before initiating the MI intervention, and the two interventions included a visual imagery exercise, a relaxation protocol, and mental preparation. MI training was directed by stepwise guidance, and detailed instructions regarding the methods were given to the children. The MI training contents could be changed based on the participant’s weaknesses or additional motor skills. Details regarding the task environment (location) were not reported. Each intervention involved a 60-min session conducted once a week for 5 weeks (total 300 min) in two studies; 45-min sessions were conducted per week for 9 weeks (total 405 min) in one study. One of the reports included the MI intervention time, which was 20 min, including video observation and actual practice.

**Table 2:** Overview of extracted motor imagery training sessions (MITS) elements.

MITS element	Wilson et al. (2002)	Wilson et al. (2016)	Adams et al. (2017)
Position	Task-specific	Task-specific	NSt
Location	NSt	NSt	NSt
Focus	Motor-focused	Motor-focused	Motor-focused
Order	MI before, between, and after PP	MI before, between, and after PP	MI before and after PP
Integration	Embedded	Embedded	Added
MI instructions medium	Acoustic and visual (CD-ROM, video)	Acoustic and visual (CD-ROM, video)	Acoustic and visual (video)
Instruction mode	Pre-recorded	Pre-recorded	Pre-recorded
Supervision	Supervised	Supervised	Supervised
Directedness	Directed with stepwise guidance	Directed with stepwise guidance	Directed
Instruction type	Detailed	Detailed	Detailed
Instruction individualization	Standardized	Standardized	Standardized
Familiarization	Received familiarization	Received familiarization	Received familiarization
Change	Changed	Changed	Changed
MI session	Individual	Individual	Individual
Eyes	Opened	Opened	Opened
Perspective	Internal and external	Internal and external	Internal and external
Mode	Kinesthetic and visual	Kinesthetic and visual	Kinesthetic and visual

## Discussion (Neurorehabilitation Study)

Our research question was aimed at examining how MI interventions are performed in children with DCD. The purpose of our literature review was to answer this question and explore the current approaches to MI intervention in children with DCD. Our literature search focused on identifying medical treatments based on the clinical diagnosis of DCD.

This is the first report to clarify the methodology of MI intervention for children with DCD. As a result of the investigation, we found that participants’ attitudes during MI were task-specific. Participants received linguistically standardized explanations, and MI was performed from the kinesthetic and visual modes from the internal (first person) and external (third person) perspectives. Participants observed and prepared videos on motor skills before starting MI, which was performed before PP and alternately during or after PP. In some ways, it was confirmed that MI interventions for healthy children and adults and children with DCD differed in several ways.

Regarding the timing of implementing MI, Feltz and Landers (1983) recommend that it be implemented before PP. Schuster et al. (2011) on the other hand, recommend that it be performed after the PP. The timing of the MI depends on the purpose of the training, such as whether the content of the imagined exercise is new learning or preparation for an acquired motion. As MI intervention for children with DCD aims to enhance weak and unacquired movements, MI was performed before PP in all studies or added during or after the PP. In other words, the afferent information obtained from the actual movement is useful for movement expression during MI.

In all studies, children observed a video of the motions before the MI intervention. This is useful for learning the motor element of the imaged motion and enables participants to prepare for MI. In addition to the PMC and the parietal lobe, which are reported to be active in MI, the brain regions that are active in action observation (AO) include the occipital lobe and the IFG (Hardwick et al., 2018). Possibly the AO can activate a wide range of brain areas with reduced activity. Recent studies suggest that combining or simultaneously using AO and MI has a better effect on exercise outcomes than MI or AO alone (Eaves et al., 2016). It has also been verified that MI and AO are more effective than MI alone in interventions for children (Scott et al., 2019). It is also reported that MI intervention causes a temporary deterioration in motor performance due to mental fatigue caused by repeated MI (Rozand et al., 2016). These findings suggest that performing MI and AO simultaneously may reduce mental stress. Also, the speed of the video to be observed is added to the nominal, and the slow-motion is used for observation. It has been reported that AO in slow motion promotes greater activation of the M1 compared to that at normal speed (Moriuchi et al., 2014, 2017). It is believed that slow-motion makes it possible to decompose and better understand the elements of motion, which in turn better activates the AO network. So far, it is known that motor-related areas, which are important for motor activity, are activated more by kinesthetic images, and the visual cortex, which processes visual information, is activated more by visual images (Guillot et al., 2009). Therefore, for the acquisition of motor skills, MI intervention often uses a first-person and kinesthetic image (Ridderinkhof and Brass, 2015). However, even if the participants are instructed to perform only the kinesthetic motor image task, there is no guarantee that they will be able to recall the pure kinesthetic motor image as instructed. Therefore, it is considered useful to use the first-person and third-person actions observed in the video in advance for MI.

## General Discussion

Brain function in children with DCD can be plastically altered and need not remain constant throughout life. A report by Williams et al. suggests that aging changes the network of pathways important for motor planning, control, and cognition and that various experiences during growth can help to develop compensatory pathways (Williams et al., 2017). A study of motor learning in healthy adults also confirmed a decrease in connectivity from the primary motor cortex (M1) to the basal ganglia and from the supplementary motor cortex (SMC) to the M1. On the contrary, changes in connectivity enhancement from the basal ganglia to the SMC and from the dorsal motor cortex to the SMC were also observed (Ma et al., 2010; Patel et al., 2013). The main purpose of our study is to derive an effective MI intervention method based on the neuroimaging studies of DCD. There are two characteristics of MI intervention for children with DCD: (1) AO is performed before MI and exercise to learn the elements of exercise that they are not good at; and (2) Perform a visual-motor task before MI or exercise to perform mental preparation and conditioning. As has been identified, areas of decline include areas important for exercise execution and sensory integration, such as SMC and IPL, and major areas of MNS, such as IFG. These areas are consistent with the main symptoms of DCD, including reduced ability to correct with motor commands and feedback, imitation, and difficulty in motor learning. It should be noted that the pars opercularis of the IFG (BA44), children with DCD had lower activation during imitation and higher activation during observation than children with TD (Licari et al., 2015; Reynolds et al., 2015a). These results suggest that observation is more effective than imitation in children with DCD, and it may be useful to perform AO before the start of training. A review of Neuroimaging studies showed that children with DCD had reduced thalamic activity and weaker connectivity to SMC. The thalamus plays an important role in relaying somatosensory and is also involved in the correction of undoing based on sensory information. It is difficult to activate the thalamus in simulations such as MI and AO that do not involve actual movements. The thalamus is also involved in selective visual spatial attention (Wilson et al., 2002, 2016), suggesting that the visual-motor task is useful in this regard. Also, children with DCD tend to use the right brain to handle more visuospatial information to compensate for their lack of somatosensory. Therefore, we think that it may be possible to activate the thalamic pathway and promote sensory-based correction of luck by combining visual information and movement. Furthermore, we focused on the cerebellum, which is important for planning and modifying exercise such as feedforward. Especially, the cerebellar-crus-I has been reported to be involved in linguistic working memory (Marvel and Desmond, 2010), it may be activated by expressing the elements of movement and perception in words. Verbalizing the elements of motion simulated by MI and AO corrects feedforward-level errors and enables motion under appropriate motor strategies. A method similar to this idea is the task-oriented approach CO-OP (cognitive orientation to daily occupational performance). CO-OP is a cognitive movement (behavioral) approach that supports children in the process of discovery and learning to specifically work on children’s cognition and achieve the motor tasks they desire. It is characteristic that it assists in controlling behavior by verbalizing the flow of problem-solving such as goal setting, planning, execution, and self-reflection (Sangster et al., 2005). By sharing motor and sensory information obtained from AO and MI with the instructor *via* language, it may be possible to clarify the motor strategy and correct it before making an error. In some people with DCD, there was an imbalance in activity in the left and right hemispheres. IHI is believed to have spurred this situation. IHI from the contralateral to the ipsilateral motor cortex has been shown to increase during MI (Liang et al., 2014). Therefore, it may be possible to suppress the activity of the right hemisphere by increasing the activity of the left hemisphere with repeated MI. In our review, we could not find out the details of the report on task environment (location). As with the PETTLEP method, MI is recommended to be performed in a real environment, and ideally subsequent practices should be performed in a similar setting. Treatment of children with DCD has often done in hospitals and therapy rooms. We consider that children need to perform tasks in real-life situations, such as the home practice reported by Adams et al. (2017).

One of the problems in research on DCD was the coexistence of ASD and ADHD. It was found that the activity of the brain region was different when DCD alone and ADHD coexisted. To clarify the brain function specialized for the symptoms of DCD, it is necessary to establish exclusion criteria and proceed with research and review. DCD is also a more highly heterogeneous disorder than other developmental disorders. Therefore, it is necessary to study by dividing into several subtypes (classification by exercise/behavior level or brain imaging, et cetera). Also, few RCT treatises are using MI for children with DCD, and it is currently difficult to carry out a meta-analysis. MI intervention is easy to have variations and needs to be verified by a unified method by each researcher.

## Conclusion

In this review, we investigated the brain activity that is the basis of clumsiness in children with DCD. Adding to what is known from previous reports, our results indicate: (1) decreased activity of the bilateral thalamus; (2) decreased connectivity of the SM1 and the left MTG and the SM1 and the bilateral PCC and precuneus; (3) loss of superiority of connectivity of the SM1 and the cerebellum and basal ganglia in children with DCD; and (4) reduction of gray matter volume in the right SFG and MFG and lower FA and axial diffusivity in regions of white matter pathways were found in DCD. Also, we investigated an intervention methodology using MI as a neurorehabilitative technique for children with DCD. Characteristically, MI intervention was performed before, during, or after PP. Then, the motor skills were learned by performing AO before MI. MI was performed from both internal and external points of view to focus on the child’s weak motor skills and to facilitate motor learning. It was considered possible to activate the brain regions that form the neural base of DCD by using MI and AO together and performing a visual-motor task. Also, it is recommended that MI and physical practice be carried out in an environment where they operate, and the method includes self-practice at home. Furthermore, neuroimaging studies suggested that it may be useful to verbalize the exercise planning obtained by MI and AO, introspection accompanying actual movements, and the flow of problem-solving.

## Author Contributions

KI, AM, and NL reviewed the literature and discussed the contents. KI wrote the article, and SZ checked the text and created part of the figure. TK checked and revised the whole article. All authors contributed to the article and approved the submitted version.

## Conflict of Interest

The authors declare that the research was conducted in the absence of any commercial or financial relationships that could be construed as a potential conflict of interest.

## Footnotes

^1^https://www.nitrc.org/projects/bnv/
