# Supplementary material for: Neural Basis and Motor Imagery Intervention Methodology Based on Neuroimaging Studies in Children With Developmental Coordination Disorders: A Review
Source: Front Hum Neurosci. 2021 Jan 22;15:620599. doi: 10.3389/fnhum.2021.620599 (PMC7862701; doi:10.3389/fnhum.2021.620599)
Supplement: Supplementary file 1 [file Data_Sheet_1.pdf]

Supplementary Material

Supplementary Table 1 (No.1 of 6). Characteristics of the included neuroimaging studies

Authors	Year	Participants	Age (SD)	Gender	Neuroimaging	Tasks	Neuroimaging results
Querne et al.	2008	DCD 9<br>TD 10	9.9 (1.8)<br>10.0 (1.1)	2F, 7M<br>3F, 7M	fMRI	Go/no-go task	DCD = TD：ACC (BA 32), SMA (BA 6), OFC (BA 47), IC (BA 13), MFC (BA 46), IPC (BA 40), and striatum.<br>DCD < TD：Network connection between right MFC and ACC, the top-down connection from right MFC to IPC.<br>DCD > TD：Network connection between bilateral ACC and IPC, the top-down connection from left MFC to IPC.
Kashiwagi et al.	2009	DCD 12<br>TD 12	10 y, 9 mo (11.6 mo)<br>10 y, 5 mo (11.9 mo)	12M<br>12M	fMRI	Tracking	DCD < TD：left SPL (BA7), IPL (BA40), and POCG (BA2) (in the tracking and watching condition).
Zwicker et al.	2010	DCD 7<br>TD 7	10.8 (1.5)<br>10.9 (1.5)	1F, 6M<br>3F, 4M	fMRI	Fine-motor, trial-tracking task	DCD > TD：left IPL (BA 40), right MFG (BA 46), right SMG (BA 40), right LG (BA 19), right PHCG (BA 30), right PCG (BA 6), right STG (BA 41), and right cerebellar lobule VI.<br>DCD < TD：left precuneus (BA 39), SFG (BA 8), IFG (BA 47) and POCG (BA 2), right STG (BA 13).
Zwicker et al.	2011	DCD 7<br>TD 7	10.8 (1.5)<br>10.9 (1.5)	1F, 6M<br>3F, 4M	fMRI	Trail-tracing task; Day 1 and Day 5	DCD < TD activation in right IPC (BA40), LG (BA18), MFG (BA9); in left FG (BA37), IPC (BA40); in right cerebellar (crus I) and left cerebellar (lobule VI and IX).

Supplementary Table 2 (No.2 of 6). Characteristics of the included neuroimaging studies

Zwicker et al.	2012a	DCD 7<br>TD 9	10 y, 10 mo<br>(1 y, 6 mo)<br>10 y, 4 mo<br>(1 y, 7 mo)	1F, 6M<br>3F, 6M	DTI	NSt	DCD < TD：MD of the corticospinal tract.<br>DCD = TD：FA.
Debrabant et al.	2013	DCD 17<br>TD 17	9.4 (0.6)<br>9.2 (0.9)	3F, 14M<br>3F, 14M	fMRI	Motor response task (predicted or unpredicted)	DCD < TD：right DLPFC (BA 9) and TPJ (BA 40), left posterior cerebellum (crus1) (unpredictable> predictive).<br>DCD = TD (prediction>no prediction).
McLeod et al.	2014	DCD 7<br>ADHD 21<br>DCD + ADHD 18<br>TD 23	13.0 (2.5)<br>12.5 (2.9)<br>11.5 (3.0)<br>11.3 (2.8)	2F, 5M<br>1F, 20M<br>4F, 14M<br>12F, 11M	fMRI	Rest	DCD < TD：bilateral IFG, IC, STG and caudate, right FOC, SG, nucleus accumbens, pallidum and putamen (functional connectivity with left M1).<br>DCD > DCD + ADHD：bilateral caudate, anterior STG, left PC, POCG, FC, right IFG, POC
Langevin et al.	2014	DCD 9<br>ADHD 27<br>DCD + ADHD 23<br>TD 26	12.2 (2.7)<br>11.8 (3)<br>11.4 (2.9)<br>11.6 (3.2)	2F, 7M<br>3F, 24M<br>4F, 19M<br>12F, 14M	DTI	NSt	DCD < TD：FA in the left lateral SLF III and bilateral SPP.<br>DCD = TD：MD of the all tracts.<br>Various differences in CT were confirmed between DCD + ADHD and TD, DCD or ADHD.

Supplementary Table 3 (No.3 of 6). Characteristics of the included neuroimaging studies

Langevin et al.	2015	DCD 14<br>ADHD 10<br>DCD + ADHD 10<br>TD 14	9 y, 9 mo<br>(1 y 7 mo)<br>9 y, 9 mo<br>(1 y 3 mo)<br>9 y, 7 mo<br>(2 y 3 mo)<br>11 y, 9 mo<br>(3 y)	9F, 5M<br>4F, 6M<br>2F, 8M<br>6F, 8M	MRI	NSt	DCD < TD：right MOC (BA 11 *), right TP was reduced in DCD.
Licari et al.	2015	DCD 13<br>TD 13	9.6 (0.8)<br>9.3 (0.6)	13M<br>13M	fMRI	Finger sequencing and hand clenching tasks	DCD < TD：the left SFG (BA9) and IFG (BA44) (finger sequencing task). DCD > TD：right POCG (BA3) (finger sequencing task). DCD = TD (hand clenching task).
Reynolds et al.	2015a	DCD 14<br>TD 12	10.1 (1.3)<br>10.1 (1.2)	14M<br>12M	fMRI	Observing, executing and imitating a finger sequencing tasks	DCD < TD：bilateral PCG (BA 6), right pars opercularis of the IFG (BA 9) and precuneus (BA 31), left MTG (BA 19), left PC (BA 30) (observation > baseline condition), pars opercularis (BA 44) (imitation).<br>DCD ＞ TD：pars opercularis (BA 44) (observation).<br>DCD = TD (action execution and imitation conditions).
Debrabant et al.	2016	DCD 21<br>TD 20	9 y, 2 mo<br>(10 mo)<br>9 y, 4 mo<br>(7 mo)	3F, 18M<br>4F, 16M	DTI	NSt	DCD < TD：left retrolenticular limb of the internal capsule (decrease mean FA together with an increase in mean RD), right retrolenticular limb of the internal capsule (borderline FA and a significantly higher RD), lower FA in sensorimotor tracts.<br>DCD < TD：the nodal efficiency at the cerebellum lobule VI and the right parietal superior gyrus.

Supplementary Table 4 (No.4 of 6). Characteristics of the included neuroimaging studies

Caeyenberghs et al.	2016	DCD 11<br>ASD 15<br>DCD + ASD 8<br>TD 19	8.8<br>9.4<br>9.8<br>9.7	11M<br>1F, 14M<br>8M<br>11F, 8M	MRI	NSt	DCD > TD：right lateral OFC (BA 11).
McLeod et al.	2016	DCD 6<br>ADHD 19<br>DCD + ADHD 14<br>TD 21	13.0 (2.8)<br>12.4 (3.1)<br>11.3 (3.8)<br>11.0 (2.8)	1F, 5M<br>1F, 18M<br>3F, 11M<br>11F, 10M	fMRI	Rest	DCD or ADHD > TD：left SM1 and left thalamus and right cerebellum (lobule V) (Connectivity).<br>DCD < TD or ADHD：right SM1 and right putamen. (DCD：right putamen was equally connected to right and left SM1.)<br>DCD + ADHD < DCD, ADHD, TD：left SM1 and bilateral precuneus, MFG and inferior lateral OC. These regions were equally connected to left and right SM1 in typically developing children and children with only ADHD or DCD.
Biotteau et al.	2017	DCD 16<br>DD 16<br>DCD + DD 16	9.6 (1.7)<br>10.3 (1.3)<br>9.9 (1.1)	4F, 12M<br>7F, 9M<br>6F, 10M	fMRI	Finger tapping sequence task (primary and learned)	DCD > DD：bilateral CG (BA31 and BA24), SMC (BA4 and BA3), PMC (BA6), TPJ (BA40, BA41, BA42, BA43, BA44, and BA22), right IC (BA 13), anterior cerebellum, left thalamus (Overt Training).<br>DCD > DD：bilateral CG (BA31 and BA24) and thalamus, right caudate and claustrum (Novel Training).<br>DCD > DCD + DD： bilateral PCG (BA4), TPJ (BA7, BA21, BA22, BA31, BA37, BA41, BA42, BA43), right CG (BA24, BA31, BA32) and anterior and posterior cerebellum, left PMC (BA6), thalamus and globus pallidus (Overt Training).<br>DCD > DCD + DD：right CG (BA31 and BA23) (Novel Training).

Supplementary Table 5 (No.5 of 6). Characteristics of the included neuroimaging studies

Reynolds et al.	2017	DCD 22<br>TD 22	9.9 (1.1)<br>9.7 (1.0)	22M<br>22M	VBM	N/A	DCD < TD：MFG (BA6, BA8), SFG (BA6) (right lateralized reductions in grey matter volume).
Thornton et al.	2018	DCD 9<br>ADHD 20<br>DCD + ADHD 18<br>TD 20	13.6 (2.7)<br>12.4 (2.8)<br>10.9 (2.6)<br>10.6 (2.8)	3F, 6M<br>2F, 18M<br>3F, 15M<br>12F, 8M	fMRI	Go/no-go task	DCD =TD：(go/ no-go task).<br>ADHD + DCD < TD：right PCG (BA4, BA6) and POCG (BA3), left SFG (BA48), MFG (BA9) and MSFG (BA32).
Reynolds et al.	2019	DCD 10<br>TD 9	10.2 (1.3)<br>10.4 (1.2)	10M<br>10M	fMRI	Finger adduction/abduction task	DCD = TD ：IPL, STS, PMv (BA6), IFG (BA46) (MNS area).<br>DCD < TD： bilateral thalamus, right caudate, and PC (BA29) (imitation).<br>DCD < TD：bilateral insula (BA13), MFG (BA6), right caudate, thalamus, left PHCG (BA30), POCG (BA3) (execution).<br>DCD < TD：bilateral caudate, right thalamus, left STG (BA41), cingulate gyrus (BA24) (motor imagery).<br>DCD < TD：bilateral precuneus (BA7), left cingulate gyrus (BA 23), TTG (BA41) (observation).

Supplementary Table 6 (No.6 of 6). Characteristics of the included neuroimaging studies

Brown-Lum et al.	2020	DCD 31<br>TD 30	10.1(1.2)<br>9.9(1.4)	5F, 26M<br>9F, 21F	DTI	NSt	DCD < TD：lower FA in corticospinal tract, cerebral peduncle, superior cerebellar peduncle, external capsule, SLF and splenium of the corpus callosum.<br>DCD < TD：lower axial diffusivity in corticospinal tract, cerebral peduncle, posterior thalamic radiation at the retrolenticular part of internal capsule and external capsule.
Riant et al.	2020	DCD 35<br>TD 23	9.8 (1.6)<br>9.9 (1.4)	8F, 27M<br>8F, 15M	fMRI	Rest	DCD < TD：bilateral SMC and PCC (BA23, BA31) and precuneus (BA7, BA31), SMC and left pMTG (functional connectivity).

SD = Standard deviation; DCD = Developmental coordination disorder; ADHD = Attention-deficit hyperactivity disorder; DD = Developmental dyslexia; ASD = Autism spectrum disorder; TD = Typical development; F = Female; M = Male; NSt = Not stated; fMRI = Functional magnetic resonance imaging; DTI = Diffusion tensor imaging; VBM = Voxel-based morphometry; BA = Brodmann area; ACC = Anterior cingulate cortex; SMA = Supplementary motor area; OFC = Orbitofrontal cortex; MFC = Middle frontal cortex; IPC = Inferior parietal cortex; SPL = Superior parietal lobule; IPL = Inferior parietal lobule; POCG = Postcentral gyrus; MFG = Middle frontal gyrus; SMG = Supramarginal gyrus; LG = Lingual gyrus; PHCG = Parahippocampal gyrus; PCG = Precentral gyrus; STG = Superior temporal gyrus; IFG = Inferior frontal gyrus; FG = Fusiform gyrus; MD = Mean diffusivity; FA = Fractional anisotropy; DLPFC = Dorsolateral prefrontal cortex; TPJ = Temporo-parietal junction; SLF = Superior longitudinal fasciculus; SPP = Superior posterior parietal; MOC = Medial orbitofrontal cortex; TP = Temporal pole; IC = Insular cortex; FOC = Frontal operculum cortex; SG = Supramarginal gyrus; M1 = Primary motor cortex; MTG = Middle temporal gyrus; PC = Posterior cingulate; CG = Cingulate gyrus; STS = Superior temporal sulcus; PMC = Premotor cortex; SMC = Sensorimotor cortex; OC = Occipital cortex; AFD = Apparent fiber density; MSFG = Medial superior frontal gyrus; MNS = Mirror neuron system; PC = Posterior cingulate; PMv = Ventral premotor cortex; TTG = Transverse temporal gyrus; POC = Parietal operculum cortex; PCC = posterior cingulate cortex; * = BA was not listed; therefore, we assigned it.

Supplementary Table 2. MITS elements

MITS element	MITS element description and categories	PETTLEP
Position	Describes the position during MI practice as task-specific or not task-specific.	Physical
Location	Describes the location during MI as task-specific or not task-specific.	Environment
Focus	Focus of the intervention classifies the main focus of task-related activities that had to be imagined: motor, strength, or cognitive.	Task
Order	Describes temporal order of MI and PP trials. MI trials could have been performed before, between, or after PP.	Timing
Integration	Describes whether MI practice has been added to PP or embedded into PP.	
MI instructions medium	MI instructions can be provided differently through one or more media types. Media type was scored as written, acoustic, or visual.	Learning
Instruction mode	In addition to the instruction medium, the mode was classified as live or pre-recorded (for example, using CD-ROM or video).	
Supervision	MITS could have been supervised or not supervised by an instructor present during the training.	
Directedness	MITS could have been directed or non-directed bases on whether stepwise guidance was provided or not.	
Instruction type	The description of MI instructions varied. Instructions could involve detailed descriptions for each part of the task that had to be imagined, simple keywords, or coarse overall MI instructions.	
Instruction individualization	MI instructions could have been individualized to the participant's problems with the task that had to be imagined (tailored), or could have been the same for each participant (standardized).	
Familiarization	Describe whether study participants had received an MI familiarization session before the MI.	
Change	Indicated whether modification of content, duration, or dosage of the MI training occurred to facilitate the learning process during the MI intervention period.	
MI session	MITS could have been classified as group sessions or as individual sessions with one participant only.	Emotion
Eyes	During the MI, the participant's eyes could have been closed or open.	
Perspective	During the MI, participants could have imagined the task from an internal (first person) or external (third person) perspective.	Perspective
Mode	During the MI, participants could have used a kinesthetic or visual mode.	

MITS = Motor imagery training session; MI = Motor imagery; PETTLEP = Physical, environment, timing, task, learning, emotion, perspective; PP = Physical practice
